# The socioeconomic impact of the COVID-19 lockdown on families affected by childhood respiratory illnesses in Cape Town, South Africa

**DOI:** 10.1371/journal.pgph.0003020

**Published:** 2024-03-28

**Authors:** Michaile G. Anthony, Graeme Hoddinott, Margaret Van Niekerk, Isabelle Dewandel, Carla McKenzie, Carien Bekker, Helena Rabie, Andrew Redfern, Marieke M. van der Zalm

**Affiliations:** 1 Desmond Tutu TB Centre, Department of Paediatrics and Child Health, Faculty of Medicine and Health Sciences, Stellenbosch University, Cape Town, South Africa; 2 School of Public Health, Faculty of Medicine and Health, The University of Sydney, Sydney, Australia; 3 Department of Paediatrics and Child Health, Stellenbosch University, Cape Town, South Africa; PLOS: Public Library of Science, UNITED STATES

## Abstract

The COVID-19 pandemic impacted families globally, directly and indirectly. Children presenting with respiratory illnesses are affected by emerging health systems and socioeconomic changes in the COVID-19 era. We explored the socioeconomic impacts of the COVID-19 lockdown on families with a respiratory illness diagnosed in their child in Cape Town, South Africa. This study was nested in a prospective observational cohort of children presenting with respiratory symptoms presumptive of COVID-19. We conducted 21 semi-structured interviews to explore the socioeconomic impact of the COVID-19 pandemic on families with a child affected by respiratory illnesses. We used case descriptive analysis and thematically organised common and divergent experiences. We found that socioeconomic challenges in low-income communities were exacerbated: 1) loss of pre-COVID sources of income (loss of income, employment and working hours), 2) shrinking employment opportunities due to business closures and strict preventative measures, 3) family network dependence to cope with financial pressures, 4) impact on education, implicating additional pressures due to lack of resources for adequate home schooling and 5) caregivers’ mental health and wellbeing being impacted, causing stress and anxiety due to loss of income. This study shows that the COVID-19 lockdown impacted the socioeconomic aspects of families caring for a child with a respiratory illness. Care became more complicated and adversely impacted the family’s emotional well-being and health-seeking behaviour. These impacts should be more carefully considered in order to strengthen health services and global health messaging in future pandemics.

## Introduction

The COVID-19 pandemic has caused the largest global health crisis of the past century and has raised many concerns for people’s livelihoods and survival [1]. As of July 2022, in Africa, there were a cumulative total of more than 12 million COVID-19 cases and more than 250,000 COVID-19-related deaths [2].

Amidst the spread of COVID-19 globally, governments across the world enforced countrywide lockdowns to curb the spread of COVID-19 and to minimise the loss of life [3]. The first COVID-19 case in South Africa was reported on the 5^th^ of March 2020 and the country went into strict national lockdown on the 26^th^ of March 2020 –which was scaled down over time with less severe restrictions. South Africa initially implemented one of the most stringent lockdowns as compared globally, causing substantial disruption in the labour markets, with already disadvantaged workers bearing the heaviest burden [4–7]. Over 2 million jobs were lost during the second quarter of 2020 [8]. Low-income earners working in the informal sector were disproportionately affected [9], requiring people to rely on their families for material and financial support [4, 10, 11].

As a means of mitigating these challenges, vulnerable communities in many high-income countries (HICs) were given economic assistance packages [10, 12]. The South African government established the temporary 6-month COVID-19 Social Relief of Distress grant, as well as food assistance and child support grants [13]. However, the monetary value (R350/€20,14/$21,91 per grant per month) was insufficient to cover the most basic needs of families. Many families in South Africa experienced difficulties in accessing grants, difficulties withdrawing money, long queues, and unclear payment dates [10]. The standard of living in South Africa in 2020 was (R7541/ €440, $448.30 per person per month) demonstrating that the grant provided was insufficient to cover the basic needs of families [14].

Food insecurity increased from 6% to a staggering 22% during the COVID-19 pandemic [15].

School closures in South Africa resulted in a loss of learning which further widened the existing educational disparities [16–18]. Economically advantaged schools (approximately 10% of all schooling in South Africa) were able to provide students with online learning resources [19]. While children from disadvantaged schools (±90%) residing in LMICs households lacked a quiet workspace, a computer, internet access, or caregivers with the time and capacity to take on the role of homeschoolers.

The COVID-19 lockdown had far-reaching implications for the health system and other public services. Tuberculosis (TB) testing and TB notifications have been significantly affected by the COVID-19 pandemic [20]. The under-five pneumonia mortality rate is significantly higher in LMICs compared to HICs, additionally, the effect of underlying co-morbidities such as TB, HIV, malnutrition and now the impact of COVID-19 disease and pandemic with restrains on health care system could have significant implications [21].

Children most severely affected by respiratory illnesses, especially Tuberculosis, in South Africa are often already from socio-economically marginalised homes. Further, COVID-19-related changes to health services delivery and access may disproportionately affect these families. However, limited data is available on the impact of COVID-19 on the social and economic context of families of children with respiratory illnesses in LMICs. We aimed to fill this gap by describing the experiences of families providing care to a child diagnosed with a respiratory illness (including COVID-19 and Tuberculosis (TB)) in Cape Town, South Africa. Specifically, we explored 1) the economic impact of COVID-19 on these families, and 2) the socio-psychological contexts for these children through which to understand the economic impact of COVID-19.

## Methods

### Study design

We used a descriptive study design and collected in-depth qualitative data. We nested the data collection in a prospective observational cohort study–called *COVID-kids*. The study was implemented in a prospective COVID-19-specific observational cohort. Children between the ages of 0–13 years old presenting with suspected severe acute respiratory syndrome coronavirus 2 (SARS-CoV-2) illness during the COVID-19 pandemic were recruited into the study, resulting in enrolment of both SARS-CoV-2 positive and negative cases.

### Setting

The study was conducted at Tygerberg Hospital (TBH) based in the Cape Metropolitan District of the Western Cape Province in South Africa. The Cape Metropolitan District is an urban/peri-urban setting with ~4,5 million residents and >100 primary care health facilities. TBH serves over 30% of the metropolitan population (3.5 million residents) [22]. TBH serves as a secondary and tertiary-level referral hospital for paediatric infectious diseases/pulmonology and suspected COVID-19 cases. The first confirmed case in the Western Cape Province was reported on the 11^th^ of March 2020. As of July 2022, the overall number of COVID-19 cases has reached its highest at 3. 9 million confirmed cases in South Africa [23, 24]. The Western Cape registered 700,000 cases with a total 670 000 recoveries and a total of 22,000 deaths [23]. In South Africa, the healthcare system comprises three levels: Primary Health Care (PHC) which includes local clinics, community healthcare centres and district hospitals which serve as the initial access point, offering essential services like family planning, immunizations, and treatment for common diseases. The secondary level of care includes regional hospitals [25]. Referrals to higher levels occur if the patient’s needs exceed the capabilities of the current level, ultimately leading to specialized care at Provincial Tertiary Hospitals equipped with advanced facilities and expert professionals [25].

### Sample

**Fig 1:**
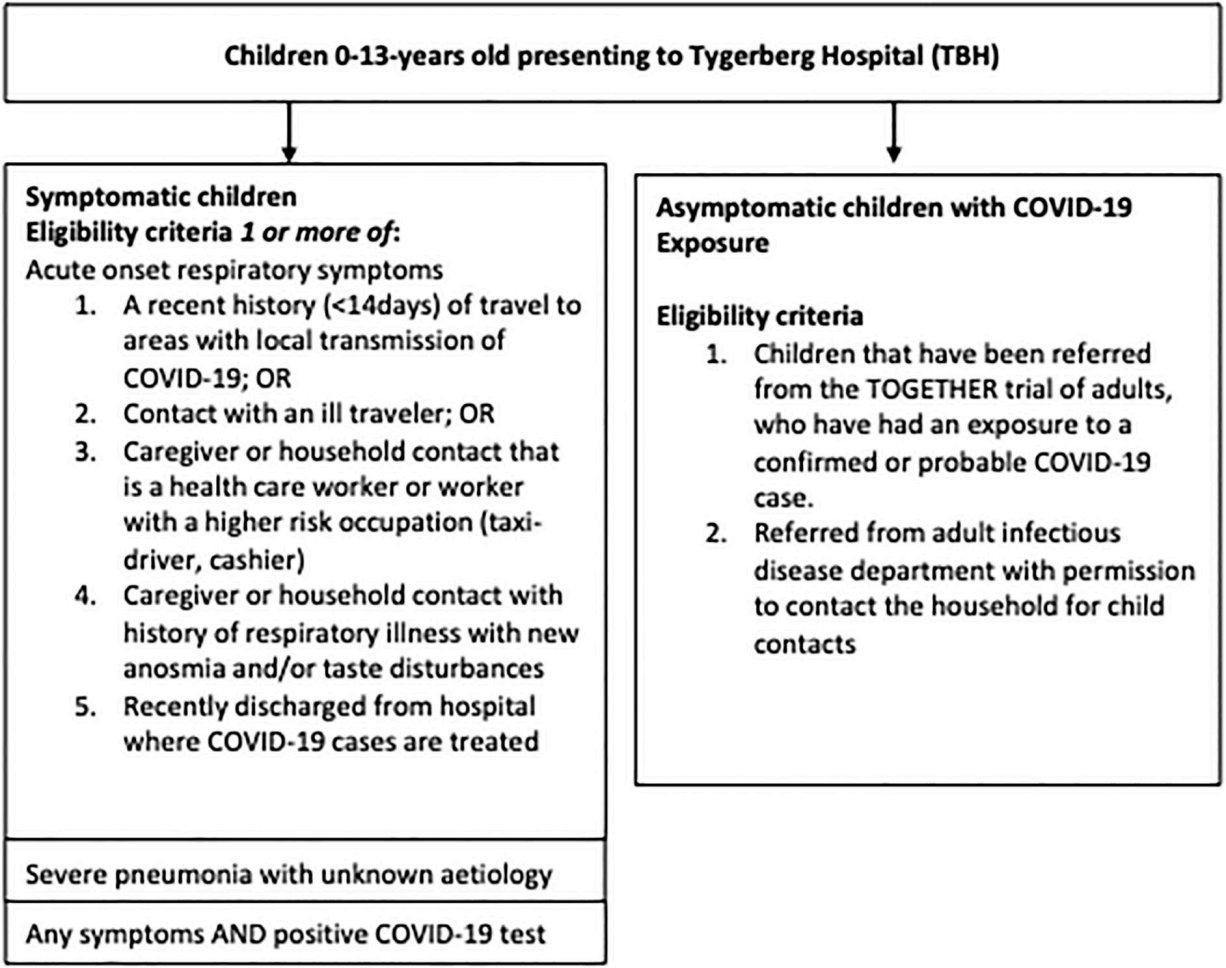
Inclusion and exclusion criteria.

We enrolled a purposive sub-sample of 21 caregivers of 20 children (⩽10-years-old) enrolled in the *COVID-kids* study (total cohort = 124) with unclear pre-enrolment diagnosis (refer to Fig 1 for inclusion and exclusion criteria). We recruited participants between November 2020 and March 2021 for this sub-study. Participants were selected for diversity in child age, sex and respiratory illness diagnosis. Five of the 20 children had confirmed SARS-CoV-2, while 15 children had other respiratory illnesses (e.g. pneumonia, respiratory syncytial virus (RSV), pulmonary tuberculosis (PTB)). We recruited participants when the child was initially hospitalised but conducted the interviews once they were receiving outpatient care. We stopped recruiting when we had reached saturation which we determined through iterative analysis of case descriptions written as field note summaries and discussed between MA, MvdZ and GH.

### Data collection procedure

We conducted semi-structured interviews with caregivers and with those children who were able to verbalise their experiences. We used a semi-structured interview guide that included participatory activities such as kinship mapping to explore the family structure and dynamics; an illness narrative and timeline activity to understand the illness journey of the participant; and a body mapping activity to explore participants’ emotional-physical-social experience. Each interview was audio-recorded, lasted for 30–90 minutes and was conducted in the participant’s preferred language (English, Xhosa or Afrikaans). At the end of each day of data collection, the data collectors (MA and TM; graduate researchers with masters-level degrees in psychology) wrote up semi-structured case summaries which were shared with the senior researchers (GH and MMvdZ) for iterative analysis.

### Data analysis

We used case comparative analysis (including verbatim transcription and translation of relevant sections) to identify the key experiences of each participant [26, 27]. We then compared these between participants and organised similar and unique experiences using principles of inductive thematic analysis. We also qualitatively compared experiences between the participants with COVID-19 and those with another respiratory illness.

### Ethical considerations

The study was approved by the Health Research Ethics Committee at Stellenbosch University and the Provincial Department of Health (N20/03/013_COVID-19). Caregivers gave signed informed consent for children’s participation. Children older than 7 years of age also signed assent. Additionally, written informed consent was obtained from the caregivers at recruitment to participate in the interviews about their experiences during the COVID-19 pandemic.

## Findings

### Characteristics of the sample

The participants in this study were the caregivers (primarily mothers (n = 19), (n = 1) father and (n = 1) grandfather) of 21 children whose ages ranged from 5 months to 10 years. The median age of the children was 12.5 months (interquartile range (6–24 months); 6 (28.6%) of whom were girls and 15 were boys (71.4%). 19 of the participants’ households included at least two children younger than 5 years old. The household sizes ranged from a minimum of 3–14 household members. Eleven participants (11/21 = 52%) reported having a caregiver or household contact that they believed placed the child at high risk for COVID-19.

### Loss of pre-COVID-19 income sources

Almost all households represented in the study experienced substantial economic challenges even before COVID-19. In many instances, there was only one family member that was working to support the whole family. Even before their child developed respiratory symptoms, all participants indicated that the COVID-19 epidemic had caused them to lose income through loss of employment and loss of shifts or working hours. Caring for their sick child further compounded the risk of income loss and made finding new income sources more difficult. For example, Joe Mxenge is a 43-year-old man and the father of a 3-year-old boy Vusi diagnosed with pulmonary tuberculosis and COVID-19. Joe told us about the well-paying job he had but due to the COVID-19 lockdown he lost his job:

“I was among the first people to be retrenched because the company was not able to pay employees. I had a well-paying job this COVID-19 thing affected us mostly in our finances and the business plan. I want to be able to provide for my children’s education even if I would die, I want them to have money to further their education”.

The loss of his job also affected his dream plan of starting a business, as he now cannot continue with his bank business loan application because he will not have money to repay the bank. The money that he currently has saved up is only assigned for emergencies and such events as he and his wife are both unemployed. The illness of Vusi further impacted the financial situation of the family:

“My wife also lost her job because she had skipped days at work to be in and out of hospital with Vusi”.

### Shrinking opportunities

Caregivers also reported the exacerbated challenge of shrinking opportunities to seek new employment due to the lockdown restrictions–especially in the restaurant industry. These shrinking opportunities arose due to the decrease in tourism, businesses and restaurant closures, employers of domestic workers and/or gardeners letting them go due to their financial difficulties and the strict (level 5 lockdown) that confined people to their homes which made it difficult to earn an income.

For example, Karabo Nene is a 21-year-old woman and the mother to Buntu a 1-year old boy diagnosed with acute renal failure. Buntu also has had a physical disability (Spastic Cerebral Palsy) since birth. Karabo lives with her uncle, three cousins and her child. Karabo described how she used to take on domestic causal jobs and would put her baby on her back while she would walk from door to door to find employment.

“I had to stay at home because I was not able to access a certain level of financial freedom because of the COVID-19 regulations and also because most companies were not hiring during the COVID-19 lockdown. I also could not work because my child was sick and had to care for him. Jobs have been impacted by COVID and not everyone is taking on people. I was not able to raise my child well, I was not able to provide for him because of COVID I was unemployed because many places were not hiring”

Many businesses closed down due to the COVID-19 lockdown restrictions which adversely impacted caregivers’ finances. In addition, caregivers working in the food and beverage industry were adversely affected as they were working fewer hours which resulted in them earning less. Sarah Ndunane a mother of a 6-month-old boy diagnosed with viral pneumonia described her experiences as follows:

“During the lockdown, restaurants were closed and were not paid as promised and now we are back from work but we have not received a back payment and we are earning less because we are working short shifts”.

### How did families survive these pressures?

Strategies employed by caregivers included depending on extended family networks to provide for their basic needs, using their savings, buying less food and/or social grants. Participants that were the breadwinners of their families were seemingly worst affected. Some caregivers reported finding alternative ways of generating an income after losing their jobs as a result of the COVID-19 lockdown regulations. However, successfully generating income this way was tenuous.

For example, Aniashe Phiri is the mother of Aneni a 10-month-old girl, Aneni was diagnosed with SARS-CoV-2/COVID-19. Anaishe explained that both she and her husband moved to South Africa to build a better life. Aniashe is from the Republic of Zimbabwe and her husband is from Malawi and they came to Cape Town in 2019. She explained that both her and her husband’s jobs were impacted by the COVID-19 lockdown.

“Before the COVID-19 pandemic we were living alone in our own place as we were able to afford it but after I lost my job, we were unable to afford the apartment and had to move in with relatives. Our shelter and food were affected by the COVID-19 pandemic. It is very painful for me not having a job as I have to ask others for money and that is why I started doing hair to make an income”.

Out of the twenty-one families included in the study, only one family received the COVID-19 social relief of distress grant. The COVID-19 social relief of distress grant was temporary assistance for individuals that were unable to meet their family’s most basic needs. Individuals receiving the social relief of distress grant were paid R350 (€20,14) per month. The grant was a short-term aid to families which were given for a period of three months. Sindy Jabavu a 43-year-old woman and grandmother to Sipho a one-year-old boy diagnosed with COVID-19 pneumonia and a history of chronic lung disease. She is the primary caregiver to Sipho and was the only caregiver that received the COVID-19 social relief of distress grant. She reported her experiences as follows:

"I received the COVID-19 relief grant [..] As I received the grant money, it had to take care of all the needs of the children, everything is dependent on it for instance to buy groceries and pay for DSTV (special TV licence) because I do not want them to miss out on lot of school program channels as a result, I was not able to buy them other things such as clothes.”

### Impact of COVID-19 lockdown on education

The COVID-19 lockdown caused disruptions in the education systems which resulted in children staying home from school and engaging in home schooling. Caregivers reported that the COVID-19 restrictions negatively impacted their children’s education due to the lockdown restrictions. Anchell Harris mother to Shane Harris a 2-year-old boy diagnosed with Croup. Anchelle lives with her husband and three children and recounted how the COVID-19 pandemic has impacted on her two older children’s schooling. She explained how the children mainly communicated with their peers and teachers through social media.

"Their schooling was affected by lockdown. The school was closed and she had to study and learn at home [..] My daughter Chrissie was communicating with other schoolmates and her class teacher via WhatsApp. The children were bored at home and were missing their friends and missed being outside"

Some caregivers reported how their education was impacted during the COVID-19 pandemic. Karabo Nene the mother of Buntu was about to start college in Johannesburg however, due to the COVID-19 lockdown and provisional travel being restricted she recounted her experiences as follows:

“When I about to go to school in January last year my child got sick and I was in hospital and I then called my father I told him that my child is sick and my father said it is fine I should stay because I will not be able to study in that condition "I’m afraid that my child might not get the same care he receives from this side or not being able to go the clinic that side because of school and I stay with only my father. The education was affected by COVID because I could not travel to that side because borders were closed.”

### Caregivers’ mental wellbeing

The caregivers in the study reported experiencing stress or anxiety as a direct result of their loss of income sources during the nationwide lockdown. For many of the caregivers, this caused worrying about money, relating to food costs as well as housing.

Nonzame Dlamini is the adoptive mother of Lisa a 5-year-old girl diagnosed with a rare form of acute myeloid leukaemia. She explained how she was worried and frustrated as she was struggling to support her family: “*I am struggling with finances because the children have different needs and food they eat*"

Additionally, caregivers’ experienced emotional and psychological difficulties in relation to the burden of care. During the COVID-19 restrictions only one caregiver was allowed in the hospital with their child. Caregivers described these experience of providing care during a worldwide pandemic and related restrictons as an emotionally demanding situation. When describing their emotions, the caregivers mentioned sadness which often resulted in crying, feeling alone and feeling depressive symptoms. One caregiver kept repeating *‘It was very depressing*. *It was very depressing’* to emphasise the emotional difficulties.

Samantha Mathews a 30-year-old woman and mother to Peter a 5-month-old boy diagnosed with COVID pneumonia. She recounted her experience of isolating at the hospital as follows:

"Sometimes I would go to the bathroom (while in hospital) and just sit there and cry [..] I felt really alone and it left me in a depressed state [..] When you are tired you get emotional and when you get emotional you become physically ill and that drains you and makes your body weak, I felt alone, I thought no one understood what I am going through [..] I felt alone like nobody cared it was almost like out of sight out of mind".

The impact of a family member losing their job had a ripple effect on the immediate family relationships. Anchelle recounted how the COVID-19 pandemic has impacted their family life especially because her husband was spending more time at home due to him losing his job. She explained how the tension in the house was unbearable at times and how her husband has become more impatient ever since he lost his job.

“My marriage was affected because my husband was at home not working and not receiving any income, that causes problems in my marriage because we were fighting a lot. We spent a lot of time together as a result we annoyed each other because the husband was always at home which is something we were not used to. He (the father of the children) would shout at the children even if they made a small mistake.

## Discussion

We found that the indirect effects of the COVID-19 epidemic (and associated non-pharmaceutical responses) introduced new stressors and exacerbated existing strains for caregivers of children with respiratory illnesses in LMICs. These stressors included the experience of financial and food insecurity, loss of individual and family livelihoods as well as emotional and psychological impacts. The indirect effects of the COVID-19 lockdown in our study went beyond immediate health concerns, encompassing economic, social, and psychological dimensions. Economically, business closures and job losses led to financial instability for many families. Socially, the lockdown induced isolation, disrupted education, and strained social support networks, while psychologically, uncertainties and prolonged restrictions increased stress and anxiety, particularly for families with children having respiratory illnesses. Furthermore, the lockdown exposed and intensified existing inequalities, disproportionately impacting vulnerable populations. Most of the participants described financial hardships, with a focus on job loss and limited financial opportunities. In high-burden, low- and middle-income settings, childhood respiratory illnesses disproportionately affect families who are economically on the margins. We show how these already very vulnerable families have been further negatively impacted by the socioeconomic costs of COVID-19.

Our findings revealed that the COVID-19 lockdown had a ripple effect on families who were already struggling before the COVID-19 pandemic. These effects impacted their finances e.g., job loss resulted in the lack of providing basic necessities such as food and shelter to their families is similar to a study conducted by [28]. The ripple effect described in our study refers to the cascading repercussions of the COVID-19 lockdown on the families in our study are indicative of a sequential series of events. Notably, instances include the progression from job loss to income deprivation, subsequently resulting in food insecurity, potential loss of shelter, or heightened vulnerability to instances of violence. Illustratively, within a specific household in our study, the principal income earner (breadwinner) experienced unemployment, precipitating intra-household conflict. This finding is similar to the study by [29] that found 48.8% of families from LMIC households experienced changes in their employment for example reduced work hours, reduced pay, loss of employment and fewer leave days. The worsening economic situation of families cannot solely be attributed to the COVID-19 lockdown measures as these disparities were already existing before the pandemic. Studies have found that people globally feared that the lockdown would cause damage to the economy and ultimately cause and/or worsen poverty and hunger among many families [30]. These sentiments particularly applied to individuals working on a contract basis and/or the informal sector that offered no protection to employees. Families in HICs received a lot of support from the government such as food assistance (food stamps, meal programmes), direct financial pay-outs of $ 1400 (R 21 779,18) to all earning below $75 000 annually (National Associations of Counties, 2021). Families in HICs also had built up financial reserves while families in our setting before the lockdown were living day to day to survive. In addition, families in South Africa only received (R350/€20,14/$21,91 per grant per month) if the recipient was unemployed from the government. A decent standard of living found that in 2020 the amount for a decent standard of living was R 7541 per person per month [14]. Thus, the R350 social relief of distress grant was insufficient for families.

South Africa has a high unemployment rate even though it is classified as an upper-middle-income country with significant inequalities and has one of the highest Gini indexes [30]. These existing inequalities worsened during the COVID-19 pandemic. The primary source of income for many South Africans is through the informal economy such as casual work which was not allowed during the hard lockdown [10, 30, 31]. This finding aligns with the sentiments echoed by the participants in our study in which many families worked in the informal sector. Domestic workers and gardeners have experienced a vast number of impacts resulting from the pandemic and related restrictions. One of the main consequences of these restrictions resulted in the loss of jobs and reduced working hours. In some instances, employers stopped paying domestic workers as a result of their financial difficulties.

The impact of the COVID-19 pandemic in South Africa as in many other countries resulted in the loss of learning due to school closures, mainly in the already disadvantaged schools leading to further widening of pre-existing education disparities [16–18]. Satisfactory online learning is nearly impossible for many disadvantaged schools and learners due to insufficient infrastructure and resources both at learners’ homes as well as in schools [19]. A survey conducted by the South African Democratic Teacher Union found that nearly two-thirds of children did not communicate with their teachers during school closures [32, 33]. However, children in our study regularly communicated with their teachers and peers via social media (WhatsApp). Disadvantaged and vulnerable learners have been disproportionately affected by the impact of COVID-19 on education as they were unable to access the resources of the education system. The COVID-19 pandemic highlighted inequalities in both the provision of resources for continued learning from home for vulnerable learners such as digital devices, data, adequate nutrition and funds as well as the disparity in how well educators, caregivers and children were equipped to do so [33].

We found that families experienced increased stress due to not being able to provide the basic necessities to their families which largely stemmed from financial losses due to unemployment, reduced income and limited job opportunities [34]. Financial insecurities (including financial difficulties, perceived job insecurity and unemployment) are associated with mental health challenges [35–38]. We found that families experienced increased stress as they were unable to provide their families with the basic necessities e.g. food, shelter etc. that directly stemmed from the financial losses due to unemployment, reduced income and limited job opportunities [30, 34]. A study among families in Nepal found that families did not have enough money to arrange meals for their families due to job losses [28]. Similarly, families in our study had to depend on external family members for basic necessities and in some cases cut on meals and ration food. Another study conducted by [39] found that families that had savings managed to sustain their families but those who did not have any savings were placed in a difficult position. Similarly, we found only one family had savings that they could tap into during the pandemic while many others did not. Interestingly, two of the families in our study started their businesses to generate income to provide for their families.

Households from lower-income households were disproportionately affected by the pandemic due to compounding the effects of already high gender-based violence, rising unemployment, inequality and poverty [40]. Young parents especially those working experienced difficulties in coping with the COVID-19-related effects. Similarly, caregivers in our study experienced mental health difficulties such as stress, anxiety, worry, fear etc. especially those who lost their jobs [31]. Caregivers’ emotional and psychological impact of COVID-19 in terms of their employment and described feeling ‘held back’, ‘big adjustments and dreams being put ‘on hold’. These sentiments were accompanied by feelings of frustration, stress, worry and anxiety. Our study suggests two recurring stressors experienced by caregivers (1) finding employment and 2) the ability to provide necessities which directly impacted on the mental well-being of caregivers. These findings show the need for mental health interventions that specifically focus on coping with the indirect impact (e.g. mental health) of pandemics are required and needs to be prioritised [31, 41–43].

Studies conducted in South Africa found a strong correlation between food insecurity and psychological distress [40]. Similarly, many of the caregivers in our study experienced emotional and psychological difficulties. However, despite these challenges, caregivers in our study remained resilient and accepted the ‘new normal’. Economic hardship, food insecurity and parenting stress increase the risk of violence such as GBV [44–47]. Many South Africans experience financial hardships and food insecurity during the pandemic due to reduced working hours and job loss, which may worsen an already stressful household situation. Similarly in our study, two caregivers reported experiencing GBV due to the job loss of their partners and the frustration of being confined [44]. The experiences of violence increased during the COVID-19 pandemic and the most common forms were verbal abuse followed by sexual harassment [48].

A strength of the study is that it explored the experiences of families caring for children with respiratory illnesses residing in an LMIC. Another strength of this study is that it led to a further understanding of the impact of the COVID-19 pandemic on families and provides insight into the effects of the lockdown measures on South African families. Limitations of this study include the small sample size and the fact that the study was only conducted in Cape Town, South Africa, limiting generalizability. Our study does not represent the experiences of children with respiratory illnesses who either did not seek care at all or who sought care at only primary or secondary care facilities. However, we believe that the core ideas that a pandemic and its responses have multiple, compounding impacts on children and their families is robust. Our data can be used to explore this further in future studies. Lastly, we did not explore the ability of participants to access the unemployment insurance fund (UIF), which was an option to get monetary government support and which could have partially mitigated some income loss.

This study provides insight into the gaps in the government’s response to the COVID-19 pandemic and how families that were already struggling financially from South Africa were disproportionately affected by the pandemic. This study can assist policymakers and the government in working on strategies to support families from South Africa and other LMICs when faced with a pandemic. In addition, this study highlights the gaps in mental health service provision for families in crisis. Mental health interventions as well as support services are required as they could assist in providing individuals and their families with coping skills. These services will not solve the problems related to job loss and unemployment but they could help individuals cope while they embark on a journey to secure employment. This study revealed some gaps in the government’s strategy to support vulnerable families during an outbreak like the COVID-19 pandemic. Further research is needed about the emotional and psychological impacts of pandemics on families and there is a need to develop strategies to enhance family’s well-being. The indirect impact of a future pandemic should be more carefully considered including global support for relief packages to support families living in South Africa and other LMICs. These interventions are not only relevant to the COVID-19 pandemic but also more generally, in the context of South Africa with persistently high rates of unemployment and social ills such as GBV. Policymakers are advised to consider the interconnected cascade of effects from our study when addressing future pandemics, acknowledging the catalytic role of job loss in triggering adversities such as income loss, food insecurity, potential homelessness, and increased household violence. Formulating comprehensive responses is essential, incorporating targeted measures like social support mechanisms, employment retention initiatives, financial assistance programs, and conflict prevention measures. Tailored policies should be informed by a nuanced understanding of specific vulnerabilities within households, as demonstrated by the experiences of primary income earners in our study, necessitating a holistic approach to effectively mitigate these indirect effects across diverse sectors while simultaneously addressing the direct health impacts of pandemics.

## References

[1] Arndt C, Davies R, Gabriel S, Harris L, Makrelov K, Modise B, et al. Impact of Covid-19 on the South African economy. Sa-Tied 2020:27.

[2] Reuters [Internet]. Daily statistics in Africa 2022 [cited 2022 Oct 3]. https://www.reuters.com/graphics/world-coronavirus-tracker-and-maps/regions/africa/

[3] Greyling T, Rossouw S, Adhikari T. The good, the bad and the ugly of lockdowns during Covid-19. PLoS One 2021;16:1–18. doi: 10.1371/journal.pone.0245546 33481848 PMC7822257

[4] Casale D, Posel D. Gender inequality and the COVID-19 crisis: Evidence from a large national survey during South Africa’s lockdown. Res Soc Stratif Mobil 2021;71:100569. doi: 10.1016/j.rssm.2020.100569 36540167 PMC9756129

[5] Espi G, Leibbrandt M [Internet]. WAVE 2 6 30 September 2020 The relationship between employment history and COVID-19 employment outcomes in South Africa 2020 [cited 2022 Oct 3]. https://cramsurvey.org/wp-content/uploads/2020/09/6.-Espi-G.-Leibbrandt-M.-_-Ranchhod-V.-2020-The-relationship-between-employment-history-and-COVID-19-employment-outcomes-in-South-Africa.pdf

[6] Jain R, Zizzamina R, Budlender J, Bassier I [Internet]. The labor market and poverty impacts of covid-19 in South Africa 2020 [cited 2022 Oct 3]. https://scholar.harvard.edu/files/ronakjain/files/covid-19-southafrica.

[7] Rogan M, Skinner C. The Covid-19 crisis and the South African informal economy: “Locked out” of livelihoods and employment. Natl Income Dyn Study–Coronavirus Rapid Mob Surv 2020:1–28.

[8] Statistics South Africa [Internet] Key findings: Quarterly employment statistics June 2021 [Cited 2022 Oct 3.] http://www.statssa.gov.za/publications/P0277/P0277September2021.pdf

[9] World Bank [Internet]. South Africa Economic Update: South Africa’s Labor Market Can Benefit from Young Entrepreneurs, Self-Employment 2021 [Cited 2022 Nov 27]. Availble from: https://www.worldbank.org/en/country/southafrica/publication/south-africa-economic-update-south-africa-s-labor-market-can-benefit-from-young-entrepreneurs-self-employment#:~:text=The%20report%20suggests%20that%20entrepreneurship,jobs%20growth%20in%20the%20future.

[10] Nyashanu M, Simbanegavi P, Gibson L. Exploring the impact of COVID-19 pandemic lockdown on informal settlements in Tshwane Gauteng Province, South Africa. Glob Public Health 2020;15:1443–53. doi: 10.1080/17441692.2020.1805787 32780633

[11] Posel D, Oyenubi A, Kollamparambil U. Job loss and mental health during the COVID- 19 lockdown: Evidence from South Africa. PLoS One 2021;16:1–15. doi: 10.1371/journal.pone.0249352 33784339 PMC8009396

[12] Loayza N, Pennings SM [Internet]. Macroeconomic Policy in the Time of COVID-19: A Primer for Developing Countries [Cited 2022 Oct 3]. https://thedocs.worldbank.org/en/doc/784631586352257045-0050022020/original/NormanLoayzaMacroeconomicPolicyintheTimeofCOVID19.pdf

[13] South African Government [Internet]. Social relief of distress 2022 [Cited 2022 Oct 3]. https://www.gov.za/services/services-residents/social-benefits/social-relief-distress#:~:text=What%20do%20you%20get%3F,extended%20for%20another%20three%20months.

[14] SASPRI [Internet]. A Decent Standard of Living [cited 2022 Oct 3]. https://www.saspri.org/SASPRI/SASPRI/research/decent-living-level/index.html

[15] Nwosu CO, Kollamparambil U, Oyenubi A. Food insecurity and health outcomes during the coronavirus pandemic in South Africa 2021. Health Econ Rev 2022;12;1–16. 35723759 10.1186/s13561-022-00375-x PMC9207854

[16] Dorn E, Hancock B, Saralatsannis J, Viruleg E [internet]. COVID-19 and learning loss-disparities grow and students need help 2020 [Cited 2022 August 18]. https://www.mckinsey.com/industries/public-sector/our-insights/covid-19-and-learning-loss-disparities-grow-and-students-need-help

[17] Hanushek EA, Woessmann L [Internet]. The economic impacts of learning losses. 2020 [cited 2022 Oct 3]. Avalible from: https://www.oecd.org/education/the-economic-impacts-of-learning-losses-21908d74-en.htm

[18] United Nations [Internet]. Policy Brief: Education during COVID-19 and beyond [cited 2022 Nov 12]. https://unsdg.un.org/resources/policy-brief-education-during-covid-19-and-beyond

[19] van der Berg S, Spaull N. Counting the Cost: COVID-19 school closures in South Africa & its impact on children. 2020. S Afr J Chil Educ; 10; 1–13.

[20] Pillay Y, Pienaar S, Barron P, Zondi T. Impact of COVID-19 on routine primary healthcare services in South Africa. South African Med J 2021;111:714–9. doi: 10.7196/SAMJ.2021.v111i8.15786 35227349

[21] Ahmed S, Mvalo T, Akech S, Agweyu A, Baker K, Bar-Zeev N, et al. Protecting children in low-income and middle-income countries from COVID-19. BMJ Glob Heal 2020;5:1–3. doi: 10.1136/bmjgh-2020-002844 32461228 PMC7254117

[22] Allwood BW, Koegelenberg CFN, Irusen E, Lalla U, Davids R, Chothia Y, et al. Clinical evolution, management and outcomes of patients with COVID-19 admitted at Tygerberg Hospital, Cape Town, South Africa: A research protocol. BMJ Open 2020;10:1–6. doi: 10.1136/bmjopen-2020-039455 32868368 PMC7462165

[23] Department of Health Republic of South Africa[Internet]. Update on Covid-19 [cited 2022 Apr 2]. https://sacoronavirus.co.za/2022/04/03/update-on-covid-19-sunday-03-april-2022/

[24] Our World in Data [Internet]. Cumulative confirmed COVID-19 cases [cited 2022 Apr 3]. https://ourworldindata.org/grapher/cumulative-covid-cases-region

[25] Western Cape Government [Internet]. The future of health care in the Western Cape 2020 [cited 2023 Dec 19]. https://www.westerncape.gov.za/other/2011/12/healthcare_2020

[26] Rutakumwa R, Mugisha JO, Bernays S, Kabunga E, Tumwekwase G, Mbonye M, et al. Conducting in-depth interviews with and without voice recorders: a comparative analysis. Qual Res 2020;20:565–81. doi: 10.1177/1468794119884806 32903872 PMC7444018

[27] Legewie N. An Introduction to Applied Data Analysis with Qualitative Comparative Analysis (QCA). Forum Qual Soc Res 2013;14:1–45.

[28] Singh DR, Sunuwar DR, Shah SK, Sah LK, Karki K, Sah RK. Food insecurity during COVID-19 pandemic: A genuine concern for people from disadvantaged community and low-income families in Province 2 of Nepal. PLoS One 2021;16:1–20. doi: 10.1371/journal.pone.0254954 34288965 PMC8294479

[29] Chen CYC, Byrne E, Vélez T. Impact of the 2020 pandemic of COVID-19 on Families with School-aged Children in the United States: Roles of Income Level and Race. J Fam Issues 2022;43:719–40. 10.1177/0192513X21994153 PMC7957335 38603084

[30] Cloete A, North A, Ramlagan S, Schmidt T, Makola L, Chikovore J, et al. “… It is like it has come up and stole our lives from us” The first 21 days: A rapid qualitative assessment of how different sectors of society experienced the COVID-19 lockdown in South Africa. Soc Sci Humanit Open 2021;4:100167. 34927060 10.1016/j.ssaho.2021.100167 PMC8665353

[31] Gittings L, Toska E, Medley S, Cluver L, Logie CH, Ralayo N, et al. ‘Now my life is stuck!’: Experiences of adolescents and young people during COVID-19 lockdown in South Africa. Glob Public Health 2021;16:947–63. doi: 10.1080/17441692.2021.1899262 33750269 PMC10105067

[32] SADTU [Internet]. The challenge of going back to school–Survey 2 [Cited 2022 Oct 3]. https://sadtu.org.za/sites/deafult/files/docs/survey-full-report.pdf.

[33] Reimers FM. Primary and Secondary Education During Covid-19. Cambridge: Springer; 2022

[34] Pfefferbaum B, Carol S. Mental Health and the Covid-19 Pandemic. N Engl J Med 2020;383. 32283003 10.1056/NEJMp2008017

[35] Wang D, Shinde S, Young T, Fawzi WW. Impacts of school feeding on educational and health outcomes of school-age children and adolescents in low- and middle-income countries: A systematic review and meta-analysis. J Glob Health 2021;11:1–27. doi: 10.7189/jogh.11.04051 34552720 PMC8442580

[36] Wright L, Steptoe A, Fancourt D. Does thinking make it so? Differential associations between adversity worries and experiences and mental health during the COVID-19 pandemic. J Epidemiol Community Health 2021;75:817–23. doi: 10.1136/jech-2020-215598 33483341 PMC7830321

[37] Kim TJ, von dem Knesebeck O. Perceived job insecurity, unemployment and depressive symptoms: a systematic review and meta-analysis of prospective observational studies. Int Arch Occup Environ Health 2016;89:561–73. doi: 10.1007/s00420-015-1107-1 26715495

[38] May T, Warran K, Burton A, Fancourt D. Socioeconomic and Psychosocial Adversities Experienced by Freelancers Working in the UK Cultural Sector During the COVID-19 Pandemic: A Qualitative Study. Front Psychol 2022;12:1–11. 10.3389/fpsyg.2021.672694 PMC8823168 35145444

[39] Donga GT, Roman N V., Adebiyi BO, Omukunyi B, Chinyakata R. Lessons learnt during covid-19 lockdown: A qualitative study of south african families. Int J Environ Res Public Health 2021;18. 34886278 10.3390/ijerph182312552 PMC8657252

[40] Nguse S, Wassenaar D. Mental health and COVID-19 in South Africa. South African J Psychol 2021;51:304–13. 10.1177/00812463211001543 PMC8107260 38603189

[41] Dalglish SL. COVID-19 gives the lie to global health expertise. Lancet 2020;395:1189. doi: 10.1016/S0140-6736(20)30739-X 32222159 PMC7194526

[42] Hankivsky O, Kapilashrami A [Internet]. Beyond sex and gender analysis: an intersectional view of the COVID-19 pandemic outbreak and response 2020 [cited 2022 Oct 2022]. https://www.qmul.ac.uk/media/global-policy-institute/Policy-brief-COVID-19-and-intersectionality.pdf

[43] Kelley M, Ferrand RA, Muraya K, Chigudu S, Molyneux S, Pai M, et al. An appeal for practical social justice in the COVID-19 global response in low-income and middle-income countries. Lancet Glob Heal 2020;8:e888–9. doi: 10.1016/S2214-109X(20)30249-7 32416766 PMC7255203

[44] Dekel B, Abrahams N. ‘I will rather be killed by corona than by him…’: Experiences of abused women seeking shelter during South Africa’s COVID-19 lockdown. PLoS One 2021;16:1–15. 10.1371/journal.pone.0259275 PMC8553161 34710174

[45] Taylor CA, Guterman NB, Lee SJ, Rathouz PJ. Intimate partner violence, maternal stress, nativity, and risk for maternal maltreatment of young children. Am J Public Health 2009;99:175–83. doi: 10.2105/AJPH.2007.126722 19008518 PMC2636621

[46] Schwab-Reese LM, Peek-Asa C, Parker E. Associations of financial stressors and physical intimate partner violence perpetration. Inj Epidemiol 2016;3. doi: 10.1186/s40621-016-0069-4 27747543 PMC4771826

[47] Lucero JL, Lim S, Santiago AM. Changes in Economic Hardship and Intimate Partner Violence: A Family Stress Framework. J Fam Econ Issues 2016;37:395–406.

[48] United Nations Women [Internet]. COVID-19 and violence against women: What the data tells us[cited 2022 Nov 18]. https://www.unwomen.org/en/news-stories/feature-story/2021/11/covid-19-and-violence-against-women-what-the-data-tells-us

